# Engineering sequence and selectivity of late-stage C-H oxidation in the MycG iterative cytochrome P450

**DOI:** 10.1093/jimb/kuab069

**Published:** 2021-09-20

**Authors:** Yohei Iizaka, Ryusei Arai, Akari Takahashi, Mikino Ito, Miho Sakai, Atsushi Fukumoto, David H Sherman, Yojiro Anzai

**Affiliations:** Faculty of Pharmaceutical Sciences, Toho University, 2-2-1 Miyama, Funabashi, Chiba 274-8510, Japan; Faculty of Pharmaceutical Sciences, Toho University, 2-2-1 Miyama, Funabashi, Chiba 274-8510, Japan; Faculty of Pharmaceutical Sciences, Toho University, 2-2-1 Miyama, Funabashi, Chiba 274-8510, Japan; Faculty of Pharmaceutical Sciences, Toho University, 2-2-1 Miyama, Funabashi, Chiba 274-8510, Japan; Faculty of Pharmaceutical Sciences, Toho University, 2-2-1 Miyama, Funabashi, Chiba 274-8510, Japan; Faculty of Pharmaceutical Sciences, Toho University, 2-2-1 Miyama, Funabashi, Chiba 274-8510, Japan; Life Sciences Institute, Department of Medicinal Chemistry, Chemistry, and Microbiology & Immunology, University of Michigan, Ann Arbor, MI 48109-2216, USA; Faculty of Pharmaceutical Sciences, Toho University, 2-2-1 Miyama, Funabashi, Chiba 274-8510, Japan

**Keywords:** multifunctional cytochrome P450 enzyme, MycG, *Micromonospora griseorubida*, mycinamicin, biosynthetic engineering

## Abstract

MycG is a multifunctional P450 monooxygenase that catalyzes sequential hydroxylation and epoxidation or a single epoxidation in mycinamicin biosynthesis. In the mycinamicin-producing strain *Micromonospora griseorubida* A11725, very low-level accumulation of mycinamicin V generated by the initial C-14 allylic hydroxylation of MycG is observed due to its subsequent epoxidation to generate mycinamicin II, the terminal metabolite in this pathway. Herein, we investigated whether MycG can be engineered for production of the mycinamicin II intermediate as the predominant metabolite. Thus, *mycG* was subject to random mutagenesis and screening was conducted in *Escherichia coli* whole-cell assays. This enabled efficient identification of amino acid residues involved in reaction profile alterations, which included MycG R111Q/V358L, W44R, and V135G/E355K with enhanced monohydroxylation to accumulate mycinamicin V. The MycG V135G/E355K mutant generated 40-fold higher levels of mycinamicin V compared to wild-type *M. griseorubida* A11725. In addition, the E355K mutation showed improved ability to catalyze sequential hydroxylation and epoxidation with minimal mono-epoxidation product mycinamicin I compared to the wild-type enzyme. These approaches demonstrate the ability to selectively coordinate the catalytic activity of multifunctional P450s and efficiently produce the desired compounds.

## Introduction

Cytochrome P450 enzymes (P450s) form a superfamily of heme-containing proteins that catalyze the oxidative modification of different organic substrates (Guengerich, 2007; Sono et al., 1996; Urlacher & Girhard, 2019). Reactions catalyzed by P450s are typically associated with the biosynthesis of bioactive compounds and xenobiotic metabolism. The biosynthetic pathways of secondary metabolites in actinomycetes that produce a wide variety of bioactive compounds rely on several P450s, which contribute significantly to their structural diversity and biological activities (Cho et al., 2019; Greule et al., 2018; Rudolf et al., 2017). At least 184 types of P450s have been functionally characterized from the genus *Streptomyces*, the largest genus among the actinomycetes, and more than 70% of them relate to the biosynthesis and late-stage tailoring of secondary metabolites (Rudolf et al., 2017). As these enzymes catalyze highly regio- and stereoselective reactions that are difficult to perform by synthetic chemistry, they have been increasingly exploited for biocatalytic and chemoenzymatic applications to produce valuable compounds (Dhakal et al., 2019; Olano et al., 2010; Park et al., 2010; Rix et al., 2002). The majority of P450s involved in secondary metabolic pathways of actinomycetes catalyze a single oxidative modification on a specific substrate, while some P450s that catalyze multiple oxidative steps involving distinct reaction mechanisms have been characterized as multifunctional or iterative P450s (Carlson et al., 2011; Iizaka et al., 2021; Miyanaga et al., 2017; Zocher et al., 2011).

**Fig. 1.**
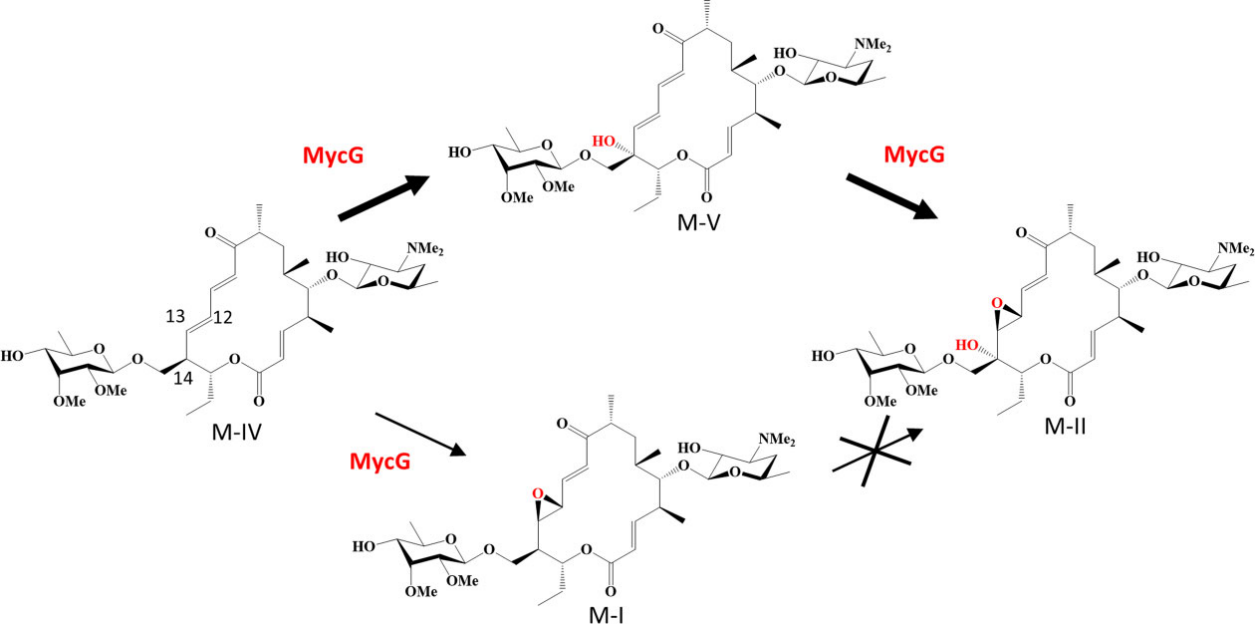
Oxidation reactions catalyzed by MycG in mycinamicin biosynthesis. The thickness of the arrow indicates the conversion efficiency of the reaction. MycG cannot catalyze hydroxylation at C-14 on M-I.

Mycinamicin is a 16-membered macrolide antibiotic produced by *Micromonospora griseorubida* A11725 that shows strong antibacterial activity against gram-positive bacteria (Satoi et al., 1980). The biosynthetic pathway of mycinamicin has been elucidated through strain mutagenesis and bioconversion studies (Anzai et al., 2004; Suzuki et al., 1990). The nucleotide sequence of the complete mycinamicin biosynthetic gene cluster consisting of 22 open-reading frames has also been determined (Anzai et al., 2003). MycG identified in this cluster is the first P450 to be characterized as a multifunctional P450 (Anzai et al., 2008, 2012). This enzyme catalyzes the sequential hydroxylation at C-14 and epoxidation at C-12/13 as the terminal mycinamicin biosynthetic steps to generate mycinamicin II (M-II) from mycinamicin IV (M-IV) (Fig. 1). M-IV is first hydroxylated by MycG to produce mycinamicin V (M-V), which undergoes sequential epoxidation to generate M-II. However, epoxidation prior to hydroxylation completely abolishes the further oxidation reactions by MycG. The iterative activity of MycG has been proposed to be associated with the translocation of substrates from the initial recognition site in two different directions by crystallography and nuclear magnetic resonance (NMR) studies (Li et al., 2012; Tietz et al., 2017). In addition, computational analysis has shown that mycinamicin I (M-I), the epoxidation product of M-IV, is not oxidatively modified by MycG because of the inherent reactivity of the substrate lacking allylic stabilization rather than the enzyme itself (Yang et al., 2020). The two methyl groups of the sugar added at C-21 on mycinamicin are also vital for substrate discrimination by MycG (Anzai et al., 2008; Li et al., 2012; Yang et al., 2020). Multifunctional P450s such as MycG offer the advantage of diversifying the structure and bioactivity of compounds because they allow multiple structural modifications with a single enzyme. However, effective utilization of these P450s demands the construction of a system for efficient production of the desired compounds by regulating individual oxidation steps.

The *M. griseorubida* A11725 wild-type strain mainly produces M-II, while M-I and M-V are produced as minor products (Anzai et al., 2012; Satoi et al., 1980). In particular, the production of M-V, an intermediate of the sequential oxidation reaction catalyzed by MycG, occurs in trace amounts. Our previous study demonstrated that the three-step oxidation reaction at the same site catalyzed by P450 RosC in the rosamicin biosynthesis of *Micromonospora rosaria* is controllable (Iizaka et al., 2020). An engineered *M. rosaria* strain reconstituted by specific amino acid residue substitutions of RosC could efficiently produce the rosamicin intermediate generated from the initial oxidation reaction. In this study, we aimed to efficiently produce M-V by protein engineering of MycG and *in vivo* genetic manipulation of *M. griseorubida*. First, MycG mutants that primarily generate M-V from M-IV were obtained from a library of MycG random mutants by screening in *Escherichia coli* whole-cell assays. We subsequently confirmed that an engineered *M. griseorubida* strain reconstituted with select MycG mutants resulted in a corresponding alteration in macrolide distribution for preferential production of M-V.

## Materials and methods

### Reagents and Enzymes

All chemical reagents were purchased from FUJIFILM Wako Pure Chemical Corporation (Tokyo, Japan), unless otherwise indicated. DNA oligonucleotides were synthesized by Eurofins Genomics (Tokyo, Japan). The DNA polymerase Prime STAR GXL (Takara Bio, Shiga, Japan) and KOD FX Neo (Toyobo, Osaka, Japan) were used for high-fidelity DNA amplification and inverse polymerase chain reaction (PCR). The Diversify PCR Random Mutagenesis kit (Takara Bio, Mountain View, CA, USA) was used to perform error-prone PCR. All DNA restriction enzymes were obtained from Takara Bio (Shiga, Japan). M-I, M-II, M-IV, and M-V were isolated and purified from the fermentation broth of *M. griseorubida* A11725 as per the procedures described by Satoi et al. (1980).

### Bacterial Strains and Media

The strains used in this study are listed in Table S1. *E. coli* strains were cultivated in Luria Bertani (LB) medium (0.5% yeast extract, 1.0% tryptone, and 1.0% sodium chloride [NaCl]). NZCYM medium (1% NZ amine, 0.5% NaCl, 0.5% yeast extract, 0.1% casamino acid, and 0.2% magnesium sulfate heptahydrate [MgSO_4_·7H_2_O], pH 7.0) was used for whole-cell assays performed with *E. coli* cells. *M. griseorubida* strains were cultured in MR0.1S medium (3.43% sucrose, 1.0% magnesium chloride hexahydrate [MgCl_2_·6H_2_O], 0.368% calcium chloride dehydrate [CaCl_2_·2H_2_O], 0.005% monopotassium phosphate [KH_2_PO_4_], 0.0004% ferrous sulfate heptahydrate [FeSO_4_·7H_2_O], 25 mM TES [pH 7.2], 0.5% tryptone, 0.5% soluble starch, 0.2% soybean meal, and 1% [vol/vol] trace element solution [Hopwood et al., 1985]). FMM medium (0.5% glucose, 0.5% calcium carbonate [CaCO_3_], 0.4% MgSO_4_·7H_2_O, 0.1% dipotassium phosphate [K_2_HPO_4_], 0.0002% cobalt chloride hexahydrate [CoCl_2_·6H_2_O], 7% soluble starch, 0.5% yeast extract, and 0.5% soybean meal) was used for the fermentation of *M. griseorubida* strains.

### Construction of Plasmids

The plasmids and PCR primers used in this study are listed in Tables S1 and S2, respectively. The MycG expression plasmid pMCGcamAB was constructed as follows: the 1.2-kb DNA fragment containing *mycG* gene was amplified by PCR using primers mycG_NdeI-F/mycG_SpeI-R and total genomic DNA of *M. griseorubida* A11725 as a template. The PCR product was cloned into the pDrive cloning vector (QIAGEN, Hilden, Germany). After confirming the sequences, the 1.2-kb NdeI-SpeI DNA fragment containing *mycG* was inserted into the P450 expression vector pCYPcamAB carrying the putidaredoxin reductase gene *camA* and the putidaredoxin gene *camB* derived from *Pseudomonas putida* (Agematu et al., 2006). Construction of plasmids to create a MycG random mutant library was performed by modifying the MEGAWHOP cloning method (Miyazaki, 2011). To introduce random mutations in the *mycG* region, the 1.2-kb DNA fragment containing *mycG* was amplified by error-prone PCR using primers mycG_RM-F/mycG_RM-R (Table S2). This PCR product was used as a megaprimer for whole-plasmid PCR amplification with pMCGcamAB. The resulting PCR product containing nicked circular plasmids was treated with DpnI and then introduced into *E. coli* Rosetta 2 (DE3) (Novagen, Merck KGaA, Darmstadt, Germany). Site-directed mutagenesis into the *mycG* region of pMCGcamAB was performed by inverse PCR using the primer pairs listed in Table S2. The accuracy of the desired mutations was confirmed by DNA sequencing.

The plasmids pMG522, pMG523, pMG524, pMG525, and pMG526 used to introduce the genes encoding the MycG mutants R111Q/V358L, W44R, V135G/E355K, V135G, and E355K, respectively, into *M. griseorubida* were also constructed by inverse PCR using the previously prepared pMG520 as a template (Anzai et al., 2012). pMG520 was constructed by inserting a 2.2-kb DNA fragment containing the region from the 3' end of *myrB* to *mycG* on the chromosome of *M. griseorubida* into the site-specific integration vector pSET152 (Bierman et al., 1992).

### Whole-cell Assays Using *Escherichia coli*

*E. coli* Rosetta 2 (DE3) transformants carrying a plasmid expressing the MycG mutant with CamA and CamB were cultivated at 32°C for 20 hr in LB medium containing ampicillin (50 μg/ml) and chloramphenicol (25 μg/ml). The seed culture (250 μl) was transferred into 25 ml of NZCYM medium supplemented with ampicillin (50 μg/ml) and chloramphenicol (25 μg/ml), and cultivated at 37°C until the OD_600_ reached 0.6–0.8. Later, 0.1 mM isopropyl-*β*-D-thiogalactopyranoside (IPTG) and 80 mg/ml 5-aminolevulinic acid were added, and the cultivation was continued at 22°C for 20 hr. The cells were harvested by centrifugation, washed with CV buffer (50 mM monosodium phosphate [NaH_2_PO_4_], 1 mM ethylenediaminetetraacetic acid disodium salt [EDTA·2Na], 10% glycerol, and 0.2 mM dithiothreitol [DTT], pH 7.4) and resuspended in CV buffer at 0.05 g/ml (wet weight). Later, 500-μl cell suspension and 0.1 mM of M-II, M-IV, and M-V were mixed and incubated at 27°C for 20 hr with vigorous shaking. The pH of the reaction mixture was adjusted to 9 using 28% ammonia solution, and mycinamicins were extracted using 2 × 500 μl ethyl acetate. The resulting organic extract was dried and dissolved in 50-μl methanol for high-performance liquid chromatography (HPLC) analysis. HPLC analysis was performed using a Hitachi Chromaster HPLC system (Hitachi High-Tech, Tokyo, Japan) comprising of a 5110 pump and a 5430 diode array detector under the following conditions: column, ODS-80TM (4.6 mm i.d. × 150 mm; Tosoh, Tokyo, Japan); mobile phase, acetonitrile/0.06% trifluoroacetic acid (30:70); flow rate, 0.8 ml/min; UV wavelength, 220 nm and 280 nm. M-I, M-II, M-IV, and M-V were quantified based on the peak areas from HPLC using peak areas derived from standard solutions. As the retention times of M-II and M-V were almost the same and the peaks overlapped, their concentrations were calculated as follows: the absorption maximum for M-II occurred at 220 nm and that of M-V was at 220 nm and 280 nm. At same concentration, M-II and M-V have equal peak areas at 220 nm. Therefore, the concentration of M-II was calculated by subtracting the concentration of M-V quantified at 280 nm from the total concentration of M-II and M-V quantified at 220 nm. All reactions were performed and analyzed in duplicates.

### Confirmation of Expressed MycG Mutants

The expression levels of MycG mutants in *E. coli* cells suspended in CV buffer at 0.05 g/ml (wet weight) during whole-cell assays were examined by sodium dodecyl sulfate polyacrylamide gel electrophoresis (SDS-PAGE). The cell suspension was sonicated using Ultrasonic Disruptor UD-201 (Tomy Seiko, Tokyo, Japan). The insoluble material was removed by centrifugation at 25,000 × g for 10 min. The protein concentration in the soluble fraction was measured using the Quick Start Bradford Protein Assay (Bio-Rad, Hercules, CA, USA) (Bradford, 1976), and adjusted to 1 mg/ml. P450 concentration in the prepared solution was determined using CO-bound reduced difference spectra (Guengerich et al., 2009). The P450 in solution was reduced by adding 10 mM sodium dithionite, and the spectrum was recorded between 400 and 500 nm as a baseline. Subsequently, CO-bound P450 was obtained by bubbling CO gas slowly through the solution for 30 s, and the spectrum was recorded between 400 and 500 nm. P450 concentration was calculated from the difference in absorbance between 450 and 490 nm with an extinction coefficient of 91 mM^−1^⋅cm^−1^ (Omura & Sato, 1964).

### Introduction of the Genes Encoding MycG Mutants into *Micromonospora griseorubida*

pMG522, pMG523, pMG524, pMG525, and pMG526 were introduced into the previously prepared *mycG* disruption mutant *M. griseorubida* TPMA0025 by intergeneric conjugation with *E. coli* S17-1 (Anzai et al., 2012). *E. coli* donor cells and *M. griseorubida* recipient cells were mixed at a ratio of 2:1 (vol/vol) and spread on MR0.1S agar plates. The plates were incubated at 27°C for 20 hr and then overlaid with 1-ml water containing nalidixic acid (0.5 mg) and apramycin (1 mg). The plates were subsequently incubated at 27°C for 2–3 weeks until the exconjugants were observed. The introduction of genes encoding MycG mutants into the resulting exconjugants was confirmed by PCR and sequence analysis.

### Analysis of Mycinamicins Produced by *Micromonospora griseorubida* Strains

To compare the levels of mycinamicins produced by each strain of *M. griseorubida*, the culture extracts were analyzed by HPLC. Each *M. griseorubida* strain was cultured in 5 ml of MR0.1 broth at 27°C on a rotary shaker at 220 rpm for 4 days. The seed culture (400 μl) was inoculated in a 500-ml Erlenmeyer flask containing 40 ml of FMM medium, and incubated at 27°C on a rotary shaker at 140 rpm for 4–14 days. One milliliter of the broth was adjusted to pH 9.0 using 28% ammonia solution and extracted with an equal volume of ethyl acetate. The organic layer was mixed with an equal volume of water at pH 2.0 using hydrochloric acid. Next, the pH of the water layer containing mycinamicins was adjusted to 9.0 using 28% ammonia solution, followed by extraction with an equal volume of ethyl acetate. After concentrating the organic solution to dryness *in vacuo*, the extracts were dissolved in 20 μl of methanol for HPLC analysis. HPLC analysis was performed under the same conditions as the whole-cell assays using *E. coli*.

## Results

### Selection of MycG Mutants that Preferentially Catalyze C-14 Monohydroxylation

**Fig. 2.**
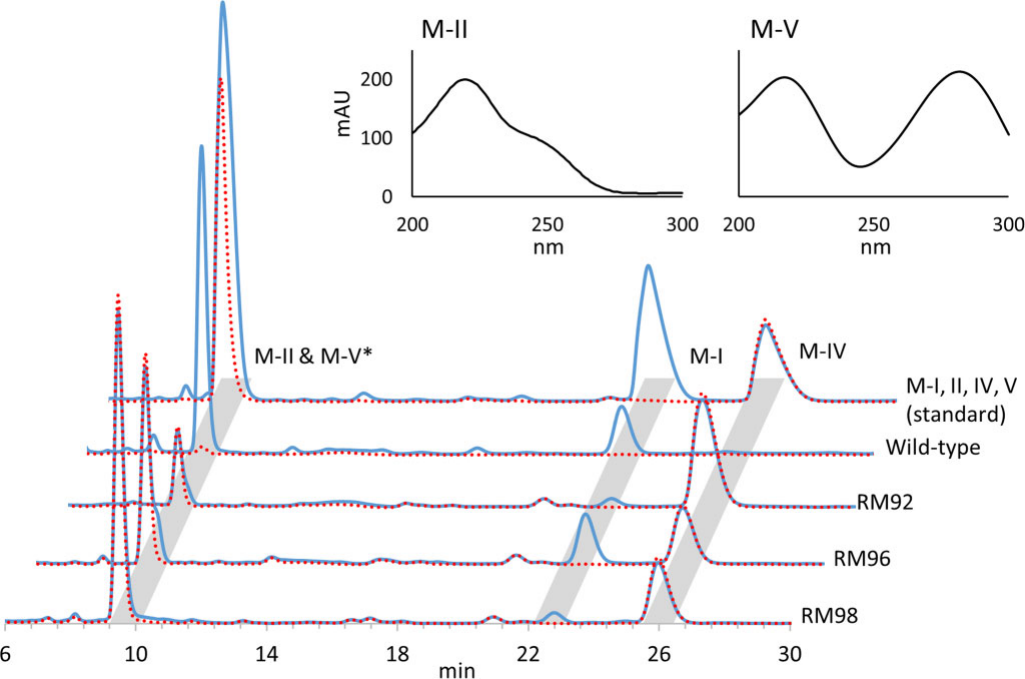
HPLC chromatograms of M-IV bioconversion with *Escherichia coli* expressing wild-type MycG and its mutants RM92, RM96, and RM98. The solid blue line represents the chromatograms at 220 nm. The dotted red lines indicate chromatograms at 280 nm. The upper panels show the UV spectrograms of M-II and M-V (1 mM). *The retention times of M-II and M-V are almost the same, and the peaks overlap. The absorption maximum for M-II occurs at 220 nm, while that of M-V is at 220 and 280 nm (upper panels). At same concentrations, M-II and M-V have equal peak areas at 220 nm. In addition, M-V has same peak areas at 220 and 280 nm. Therefore, M-V can be detected with a chromatogram at 280 nm, and M-II can be detected by subtracting the chromatogram at 280 nm from that at 220 nm.

Multifunctional P450 MycG catalyzes sequential C-14 allylic hydroxylation and C-12/13 epoxidation to generate M-II from M-IV. The M-V intermediate is generated from initial C-14 hydroxylation by MycG resulting in low-level accumulation in the mycinamicin-producing *M. griseorubida* A11725 strain. To stimulate the efficient production of intermediate products by multifunctional P450, MycG mutants that preferentially catalyze monohydroxylation were screened from a library of random *mycG* mutations. Screening was performed using previously reported *E. coli* whole-cell assays utilizing a P450 expression system (Iizaka et al., 2020). This system enables the coexpression of CamA and CamB derived from *Pseudomonas putida* as surrogate redox partners. The library of *E. coli* expressing MycG random mutants was constructed using the modified MEGAWHOP method (Miyazaki, 2011) and subjected to bioconversion with M-IV as substrate. We compared the reaction mixtures from 134 strains using HPLC and obtained three MycG mutants that showed a product profile that differed from the wild-type strain (Fig. 2). Wild-type MycG routinely converts M-IV to M-II with low levels of epoxidized metabolite M-I. MycG mutants RM92, RM96, and RM98 mainly generated M-V with some M-IV substrate remaining. The amount of M-I generated by MycG variants RM92 and RM98 was lower compared to wild-type and RM96. These results suggest that MycG mutants RM92, RM96, and RM98 contain amino acid mutations that alter the catalytic activity of MycG and favor monohydroxylation. Nucleotide sequence analysis of the plasmids encoding these MycG mutants (Table 1) revealed amino acid substitutions on MycG and localized by comparing to the cocrystallized complex of MycG and M-IV reported by Li et al. (PDB 2Y98) (2012). RM92 contains three amino acid substitutions in the D-, F-, and L-helices, while RM96 contains one amino acid substitution in the β1-sheet. RM98 possesses two amino acid substitutions in the E- and L-helices.

**Table 1:** Mutations found in the *mycG* region of the plasmids encoding MycG mutants RM92, RM96, and RM98

Mutant	Position of mutation	Nucleotide change	Amino acid change	Structure region^a^
RM92	332	G to A	R111Q	D-helix
	464	C to T	S155F	F-helix
	1072	G to T	V358L	L-helix
				
RM96	130	T to A	W44R	β1-sheet
	1026	C to T	Silent	−
				
RM98	216	T to G	Silent	−
	404	T to G	V135G	E-helix
	810	A to C	Silent	−
	1063	G to A	E355K	L-helix

^a^The region was determined from the crystal structure of MycG (PDB 2Y98).

### Identification of Amino Acid Mutations that Affect the Catalytic Activity of MycG

**Fig. 3.**
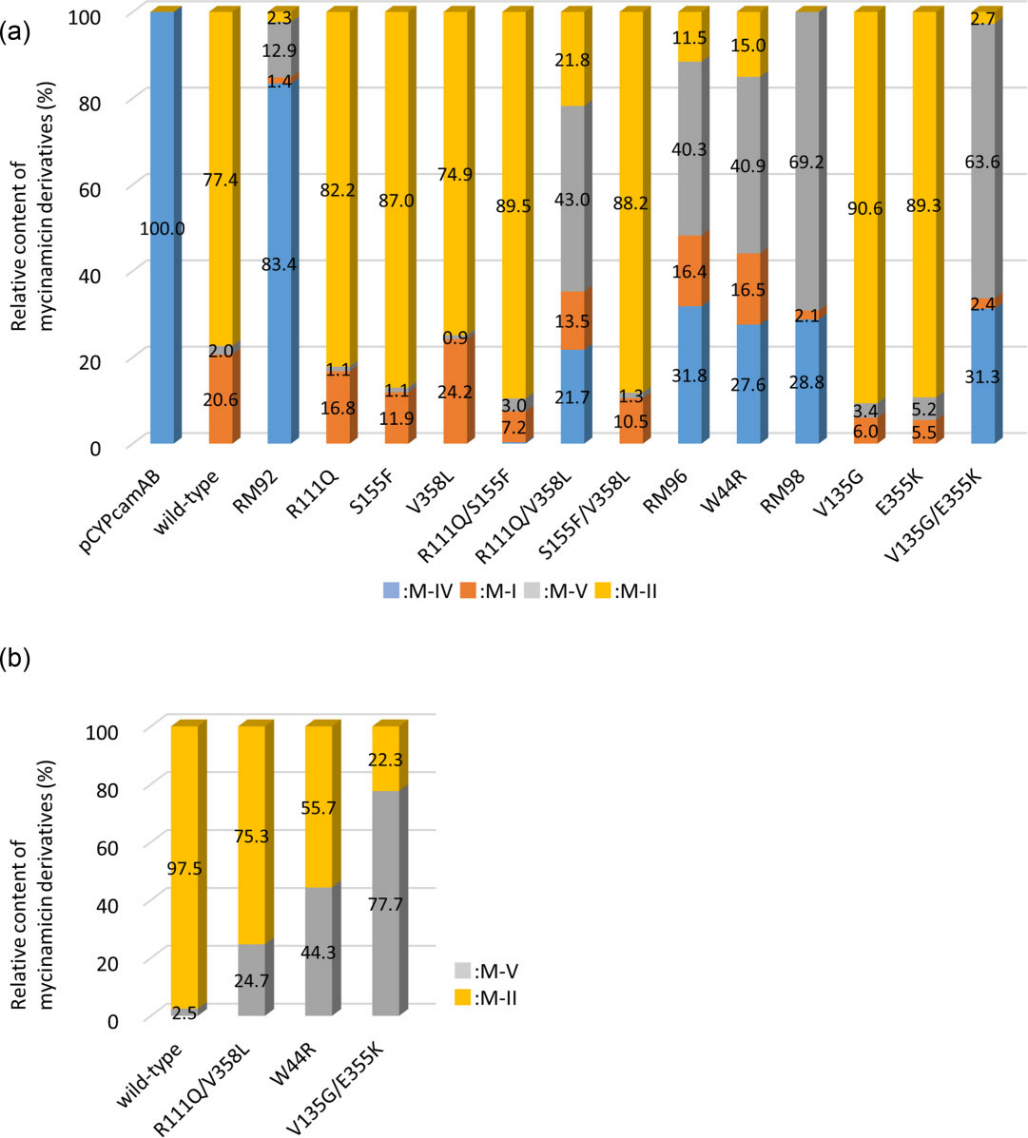
Comparison of product profiling between wild-type MycG and its mutants with M-IV (a) and M-V (b) as substrates. All reactions were performed and analyzed in duplicates. The relative content of mycinamicins was determined based on the peak area from HPLC, as described in Materials and Methods section, and represented by the average value in duplicates. The relative contents of mycinamicins for each reaction are shown in Tables S3 and S4.

*E. coli* expressing MycG mutants with single or double amino acid substitutions derived from RM92, RM96, and RM98 were prepared and subjected to whole-cell assays using individual mycinamicins as substrates (Fig. 3; Tables S3 and S4). The bioconversion rates of M-II and M-I in the reaction mixture of M-IV and wild-type MycG were 77.4% and 20.6%, respectively, while that of M-V was only 2.0% (Fig. 3a). All three MycG mutants bearing a single amino acid substitution derived from RM92 generated M-II equal to or more than the wild-type strain. The MycG double mutant R111Q/V358L showed product profiles different from RM92 (triple mutant of R111Q/S155F/V358L), but generated more M-V (43.0%) than M-II (21.8%). The MycG mutant W44R exhibited a product profile similar to the RM96 strain and generated more M-V (40.9%) than M-II (15.0%). Although MycG mutants V135G and E355K with a single amino acid substitution derived from RM98 had higher bioconversion rates to M-II than the wild-type, the double mutant V135G/E355K selectively generated M-V (63.6%). These results suggest that the R111Q/V358L, W44R, and V135G/E355K mutations altered the catalytic activity of MycG to preferentially catalyze C-H oxidation at the allylic C-14 position. In addition, the V135G and E355K mutants displayed a tendency to prioritize sequential hydroxylation and epoxidation over direct epoxidation of M-IV as compared to the wild-type strain because their bioconversion rates to M-I (5.5–6.0% conversion) were lower than the wild-type strain (20.6% conversion).

Whole-cell assays using M-V as a substrate were performed to evaluate the catalytic activity of the R111Q/V358L, W44R, and V135G/E355K mutants for conversion to M-II and display reduced catalytic activity for the terminal epoxidation step (Fig. 3b; Table S4). Wild-type MycG converted 97.5% of M-V into M-II, whereas R111Q/V358L, W44R, and V135G/E355K mutants converted 75.3%, 55.7%, and 22.3% of M-V into M-II, respectively. The direct conversion of M-V to M-II in these strains was reduced. In whole-cell assays using M-II as a substrate, no further oxidation reaction occurred in any of the samples (data not shown).

To evaluate the associations between alterations in catalytic activity and expression levels in whole-cell assays, we confirmed the expression of wild-type MycG and its mutants in *E. coli* by SDS-PAGE and CO-bound reduced difference spectra (Fig. S1; Table S5). The 44-kDa proteins presumed to represent MycG and its mutants were detected in all *E. coli* cells, except for the negative control carrying the P450 expression vector pCYPcamAB. As no difference was observed in the signal intensity, the expression level of each mutant was essentially equivalent compared to wild-type strain. However, the concentrations for R111Q/V358L, W44R, and V135G/E355K mutants based on the CO-bound reduced difference spectra were lower than 1/10 of the MycG concentration detected for the wild-type strain. These analyses suggest that R111Q/V358L, W44R, and V135G/E355K mutants induced almost no spectral change owing to CO binding and may not carry correctly folded active forms of P450s. Despite the fact that these mutants catalyze monohydroxylation to generate M-V from M-IV, it remains unclear why the CO-bound reduced difference spectra were not observed.

### Efficient Production of M-V by Mycinamicin-Producing *Micromonospora griseorubida*

*M. griseorubida* A11725 predominantly produces M-II owing to the sequential MycG-mediated hydroxylation and epoxidation of M-IV via M-V. The wild-type accumulation of M-V, an intermediate within multiple reactions catalyzed by MycG, is very low. Whole-cell assays revealed that MycG mutants R111Q/V358L, W44R, and V135G/E355K selectively generate M-V from M-IV. We then examined whether it is possible to efficiently produce M-V by *in vivo* reconstitution of these mutants in the mycinamicin biosynthetic pathway. Furthermore, MycG mutants, V135G and E355K, which showed a reduced tendency for direct epoxidation of M-IV to M-I, were also investigated in *M. griseorubida.*

The *mycG*-disruption strain *M. griseorubida* TPMA0025 and the *mycG* complementation strain *M. griseorubida* TPMA0047 were prepared as previously described (Anzai et al., 2012). The *mycG* gene in strain TPMA0025 was replaced with the disruption cassette FRT-*oriT-neo*-FRT-*attB*. TPMA0047 is a strain in which *mycG* is inserted into the chromosome of TPMA0025 by recombination between the *attP* and *attB* sites by the site-specific integration vector pSET152. We obtained *M. griseorubida* TPMA0073, TPMA0074, TPMA0075, TPMA0076, and TPMA0077 with the genes encoding the MycG mutants R111Q/V358L, W44R, V135G/E355K, V135G, and E355K, respectively (Table S1). These strains were generated by introducing site-specific mutations into the *mycG* region of the plasmid used to obtain TPMA0047, followed by the integration of the plasmid into TPMA0025.

**Fig. 4.**
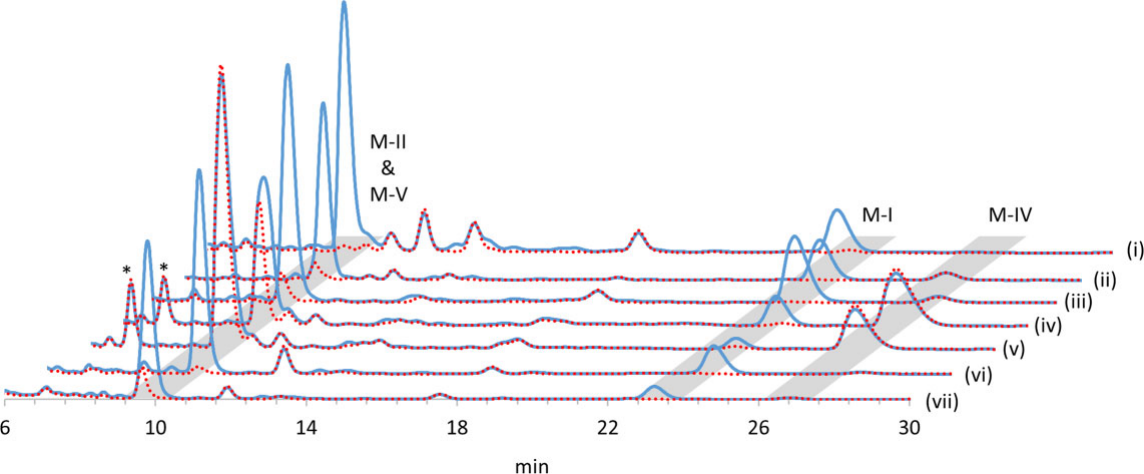
HPLC chromatograms of culture extracts obtained from *Micromonospora griseorubida* A11725 (i), TPMA0047 (ii, reconstitution of MycG wild-type), TPMA0073 (iii, reconstitution of MycG mutant R111Q/V358L), TPMA0074 (iv, reconstitution of MycG mutant W44R), TPMA0075 (v, reconstitution of MycG mutant V135G/E355K), TPMA0076 (vi, reconstitution of MycG mutant V135G), and TPMA0077 (vii, reconstitution of MycG mutant E355K). The solid blue line represents the chromatograms at 220 nm. The dotted red lines indicate chromatograms at 280 nm. The analysis conditions were the same as those shown in Fig. 2. *Peaks showing absorption maxima at 220 and 280 nm were detected in the culture extracts derived from TPMA0074 and TPMA0075. The results of liquid chromatography-mass spectrometry for these peaks were consistent with those of mycinamicin M-VI (Anzai et al., 2012). As this compound is a mycinamicin biosynthetic intermediate generated in the upper process of the biosynthetic step involving MycG, it was assumed to be unrelated to the action of MycG mutants.

To compare the production profiles of mycinamicins by strains reconstituted with the MycG mutants, culture extracts after 10 days of incubation were analyzed by HPLC (Fig. 4; Table 2). TPMA0074 and TPMA0075 produced 4.0 and 12.4 μg/ml of M-V, respectively, thus significantly enhancing M-V productivity compared to the wild-type strain A11725 and TPMA0047. In particular, TPMA0075 mainly yielded M-V, and its productivity was equal to or higher than M-II productivity by *M. griseorubida* A11725 and TPMA0047. The amount of M-V produced by TPMA0073 was almost the same as that produced by TPMA0047. The highest M-V productivity was demonstrated by TPMA0075, which was consistent with the data from whole-cell assays, wherein M-IV was reacted with *E. coli* expressing the MycG mutant V135G/E355K (Fig. 3a). Furthermore, M-V productivity in the order of TMPA0075 > TPMA0074 > TPMA0073 corresponded with the order of low bioconversion rates to M-II in whole-cell assays using M-V as a substrate (Fig. 3b). Together these results indicate that the modification of MycG with amino acid substitutions is an effective strategy for efficiently producing M-V, which normally accumulates at a very low level. The ratios of M-I to the amount of M-I, M-II, and M-V produced by TPMA0076 and TPMA0077 reconstituted with the MycG mutants V135G and E355K, were 19.0% and 9.9%, respectively. These values were significantly lower than the value for TPMA0047 (27.9%). Therefore, mutations in V135G and E355K were shown to skew the reaction selectivity of M-IV toward sequential hydroxylation and epoxidation in mycinamicin biosynthesis in *M. griseorubida*.

**Table 2:** Production of mycinamicins by *Micromonospora griseorubida* strains

		Production of mycinamicins (μg/ml)			
Strain	MyG	M-IV	M-I	M-V	M-II
A11725	Wild-type	0.0	3.0 ± 0.4	0.3 ± 0.2	11.1 ± 0.3
TPMA0047	Wild-type	1.3 ± 0.7	3.4 ± 1.0	1.1 ± 0.8	7.7 ± 1.8
TPMA0073	R111Q/V358L	0.4 ± 0.4	3.5 ± 2.1	0.6 ± 0.5	7.0 ± 4.0
TPMA0074	W44R	9.5 ± 1.2	1.7 ± 0.6	4.0 ± 0.1	4.8 ± 0.4
TPMA0075	V135G/E355K	5.7 ± 0.4	0.6 ± 0.1	12.4 ± 0.4	3.3 ± 1.0
TPMA0076	V135G	0.0	2.0 ± 0.0	0.3 ± 0.0	8.2 ± 0.4
TPMA0077	E355K	0.1 ± 0.0	0.7 ± 0.1	0.9 ± 0.0	5.5 ± 0.0

*Note:* The production of mycinamicins was determined based on the peak area from HPLC, as described in Materials and Methods section. Errors are the standard deviations of the results from duplicate experiments.

As for TPMA0075, which showed the best enhancing M-V productivity, the production of M-I, M-II, M-IV, and M-V was compared to the wild-type strain A11725 for 4–14 days to confirm the change to production profiles of mycinamicins toward culture period (Fig. S2). The production of mycinamicns by TPMA0075 became steady state after 8 days of culture. The relative content of M-I, M-II, M-IV, and M-V remained almost unchanged during longer incubation time until 14 days.

## Discussion

The current study reports the identification of amino acid residues in MycG that result in altered catalytic activity and selectivity reflected by product profile changes. Substrate recognition by MycG involves three distinct regions, the hydrophobic pocket that binds the mycinose sugar of M-IV, the active site, and the FG/BC loop that form a salt–bridge interaction with the desosamine sugar of M-IV (Yang et al., 2020). In previous study, triple mutations at L83, L94, and L227 in the hydrophobic pocket and double mutations at E237 and G281 associated with the active site essentially eliminated enzyme activity and remarkably reduced expression levels. The D226A mutation at the site involved in the formation of a hydrophobic pocket in helix I, the K80A mutation at the BC loop, the R140A mutation at the FG loop, and the S170D mutation that generates a new salt–bridge interaction with desosamine led to alterations in the conversion profiles for M-IV relative to the wild-type. Mutants carrying these mutations showed M-I and M-V productivity from M-IV as compared to the wild-type, but significantly reduced the generation of M-II from M-IV. It has been hypothesized that the change in M-II yield by these mutants is associated with the disfavored M-V binding and faster release of M-V from the active site of MycG.

We obtained two types of MycG mutants with altered product profiles using random mutagenesis and subsequent mutational analysis. MycG mutants R111Q/V358L, W44R, and V135G/E355K selectively catalyze monohydroxylation to form M-V from M-IV. The V135G/E355K mutant is especially effective in producing M-V following *in vivo* reconstitution in *M. griseorubida*. The other MycG mutants V135G and E355K display enhanced sequential hydroxylation and epoxidation to generate M-II with corresponding reduced direct epoxidation of M-IV to M-I compared to the wild-type. The five amino acid substitutions derived from these mutants occurred at sites that were structurally different from the above-mentioned three regions involved in substrate recognition by MycG (Table 1). The single mutation of R111Q in helix D and V358L in helix L had no effect on the catalytic activity of MycG, indicating that these mutations cooperatively act to alter product profiles of MycG. The product profiles of R111Q/V358L mutant were different between *E. coli* whole-cell assays and *in vivo* studies using *M. griseorubida*. This variant outcome could be due to alternative redox partners in the two expression systems. Previous studies have revealed that the functional characteristics of MycG can vary depending on the type of the redox partner (Zhang et al., 2014, 2018). Therefore, these mutations may affect the interactions with redox partners. The alterations in product profiles by mutations of W44R in the β1-sheet and V135G/E355K in helices E and L highlight the consistency between the whole-cell assays and mycinamicin-producing *M. griseorubida*. Interestingly, although the single mutations of V135G and E355K preferred the two-step oxidation pathway of hydroxylation and epoxidation over direct epoxidation of M-IV, the corresponding double mutations result in selective monohydroxylation to generate M-V. The mutation sites of W44R, V135G, and E355K were also different from the previously reported substrate recognition of MycG (Li et al., 2012). At first glance, these mutation sites appear to be unrelated to substrate recognition or catalytic properties. However, the β1-sheet of P450BM3 from *Bacillus megaterium* makes the substrate binding domain (Ravichandran et al., 1993). V135G mutation may cause a conformational change of helix I involved in substrate binding and catalytic activity because the side chain of V135 in MycG is close to helix I. E355K mutation is particularly within the heme-binding region of helix L (Hasemann et al., 1995; Mestres, 2005; Werck-Reichhart and Feyereisen, 2000), and may affect the placement of heme within MycG. We hypothesize that these factors give rise to different selectivity for oxidation of M-IV by the MycG mutants. The expression levels of R111Q/V358L, W44R, and V135G/E355K mutants whose catalytic ability was oriented toward monohydroxylation were similar to wild-type during heterologous expression in *E. coli*; however, the active P450 concentration according to the CO-bound reduced difference spectra was significantly reduced.

Over the past decade, the reactions of several multifunctional P450s derived from bacterial secondary metabolite pathways have been controlled or modified. Bifunctional GfsF catalyzes C-8/9 epoxidation followed by C-10 hydroxylation in FD-891 antibiotic biosynthesis (Kudo et al., 2010). The hydroxylation activity of this P450 is abrogated in a T300V mutant, due to elimination of a hydrogen bond with the C-7 hydroxy group of the substrate upon C-8/9 epoxidation (Miyanaga et al., 2017). AurH catalyzes allylic C-7 hydroxylation and the subsequent oxidation of the allylic methyl group at C-9a to yield the tetrahydrofuran ring in aureothin biosynthesis (He et al., 2004; Muller et al., 2006). F89W and T239F mutations in the hydrophobic pocket binding the pyrone ring of the substrate result in loss of the C-7 hydroxylation step, which activate a three-step oxidation cascade at C-9a (Zocher et al., 2011). In the present study, we achieved efficient production of M-V, an intermediate product of MycG that is difficult to produce due to its transient nature in the biosynthetic pathway to the terminal metabolite M-II. There are a certain number of multifunctional P450s involved in natural product biosynthesis. Therefore, our system for efficient production of the desired compounds by the combination of P450 protein engineering and *in vivo* genetics is expected to be useful in the field of natural product biosynthesis. Although the multistep reactions by MycG have not been completely controlled, the detailed functional analysis of MycG mutants obtained in this study will lead to further elucidation of the mechanism underlying the multifunctionality exhibited by MycG.

## Supplementary Material

kuab069_Supplemental_File
