# Supplementary material for: Engineering sequence and selectivity of late-stage C-H oxidation in the MycG iterative cytochrome P450
Source: J Ind Microbiol Biotechnol. 2021 Sep 20;49(1):kuab069. doi: 10.1093/jimb/kuab069 (PMC9113108; doi:10.1093/jimb/kuab069)
Supplement: kuab069_Supplemental_File [file kuab069_Supplemental_File.zip › kuab069.pptx]

## Slide 1
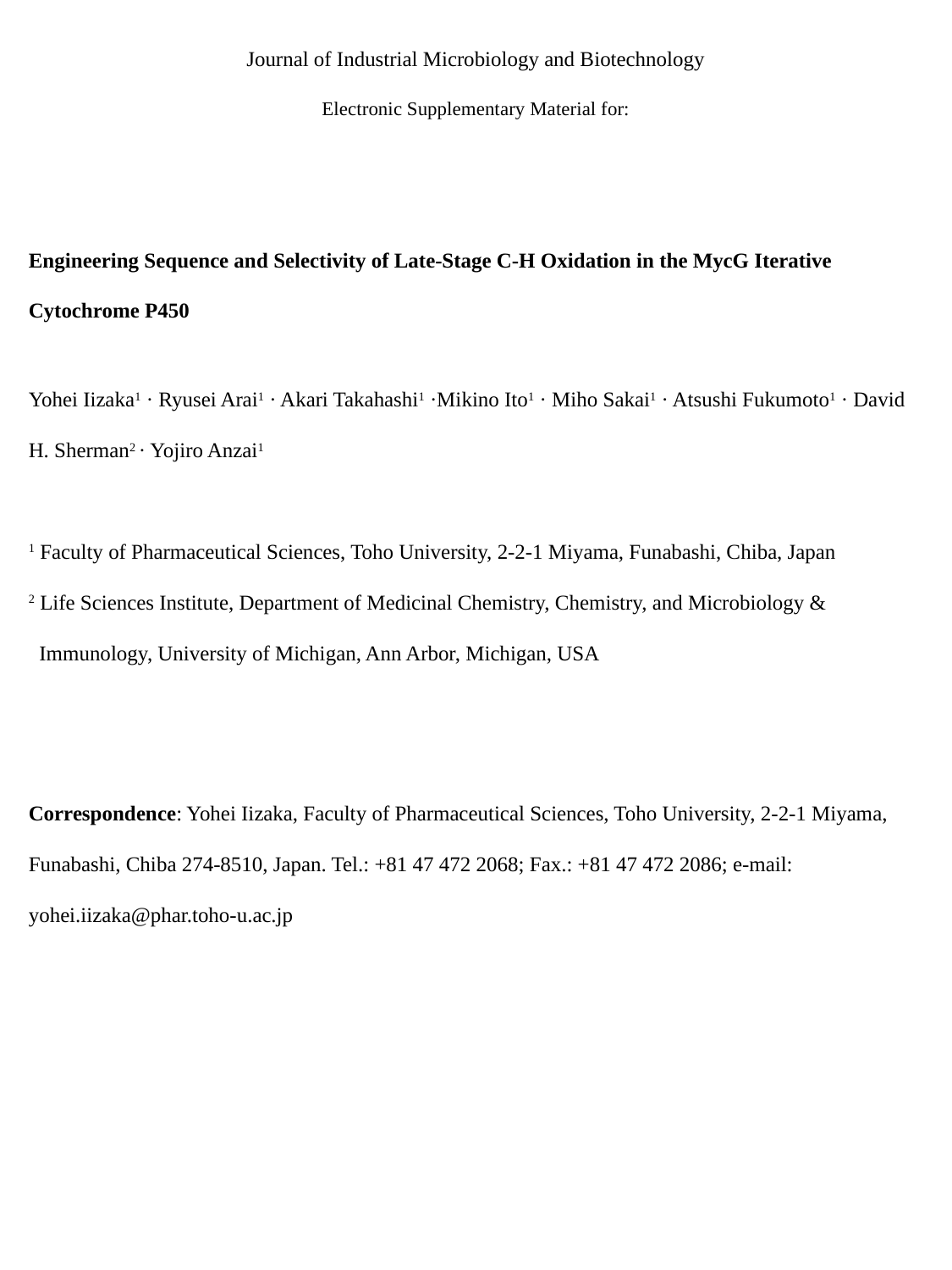

Journal of Industrial Microbiology and Biotechnology
Electronic Supplementary Material for:
Engineering Sequence and Selectivity of Late-Stage C-H Oxidation in the MycG Iterative Cytochrome P450
Yohei Iizaka1 · Ryusei Arai1 · Akari Takahashi1 ·Mikino Ito1 · Miho Sakai1 · Atsushi Fukumoto1 · David H. Sherman2 · Yojiro Anzai1
1 Faculty of Pharmaceutical Sciences, Toho University, 2-2-1 Miyama, Funabashi, Chiba, Japan
2 Life Sciences Institute, Department of Medicinal Chemistry, Chemistry, and Microbiology &
 Immunology, University of Michigan, Ann Arbor, Michigan, USA
Correspondence: Yohei Iizaka, Faculty of Pharmaceutical Sciences, Toho University, 2-2-1 Miyama, Funabashi, Chiba 274-8510, Japan. Tel.: +81 47 472 2068; Fax.: +81 47 472 2086; e-mail: yohei.iizaka@phar.toho-u.ac.jp

## Slide 2
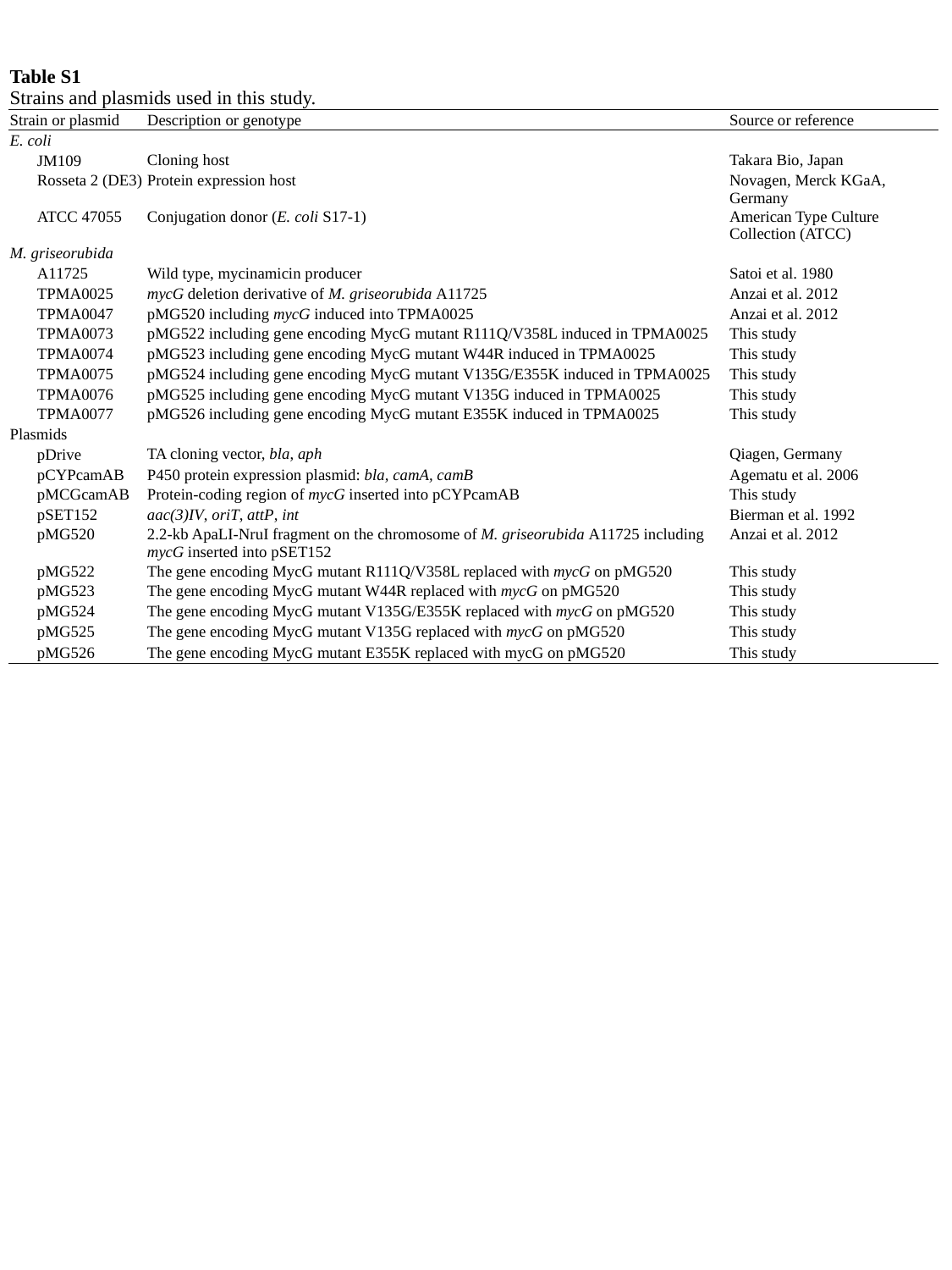

| Table S1 Strains and plasmids used in this study. | | | |
| --- | --- | --- | --- |
| Strain or plasmid | | Description or genotype | Source or reference |
| E. coli | | | |
| | JM109 | Cloning host | Takara Bio, Japan |
| | Rosseta 2 (DE3) | Protein expression host | Novagen, Merck KGaA, Germany |
| | ATCC 47055 | Conjugation donor (E. coli S17-1) | American Type Culture Collection (ATCC) |
| M. griseorubida | | | |
| | A11725 | Wild type, mycinamicin producer | Satoi et al. 1980 |
| | TPMA0025 | mycG deletion derivative of M. griseorubida A11725 | Anzai et al. 2012 |
| | TPMA0047 | pMG520 including mycG induced into TPMA0025 | Anzai et al. 2012 |
| | TPMA0073 | pMG522 including gene encoding MycG mutant R111Q/V358L induced in TPMA0025 | This study |
| | TPMA0074 | pMG523 including gene encoding MycG mutant W44R induced in TPMA0025 | This study |
| | TPMA0075 | pMG524 including gene encoding MycG mutant V135G/E355K induced in TPMA0025 | This study |
| | TPMA0076 | pMG525 including gene encoding MycG mutant V135G induced in TPMA0025 | This study |
| | TPMA0077 | pMG526 including gene encoding MycG mutant E355K induced in TPMA0025 | This study |
| Plasmids | | | |
| | pDrive | TA cloning vector, bla, aph | Qiagen, Germany |
| | pCYPcamAB | P450 protein expression plasmid: bla, camA, camB | Agematu et al. 2006 |
| | pMCGcamAB | Protein-coding region of mycG inserted into pCYPcamAB | This study |
| | pSET152 | aac(3)IV, oriT, attP, int | Bierman et al. 1992 |
| | pMG520 | 2.2-kb ApaLI-NruI fragment on the chromosome of M. griseorubida A11725 including mycG inserted into pSET152 | Anzai et al. 2012 |
| | pMG522 | The gene encoding MycG mutant R111Q/V358L replaced with mycG on pMG520 | This study |
| | pMG523 | The gene encoding MycG mutant W44R replaced with mycG on pMG520 | This study |
| | pMG524 | The gene encoding MycG mutant V135G/E355K replaced with mycG on pMG520 | This study |
| | pMG525 | The gene encoding MycG mutant V135G replaced with mycG on pMG520 | This study |
| | pMG526 | The gene encoding MycG mutant E355K replaced with mycG on pMG520 | This study |

## Slide 3
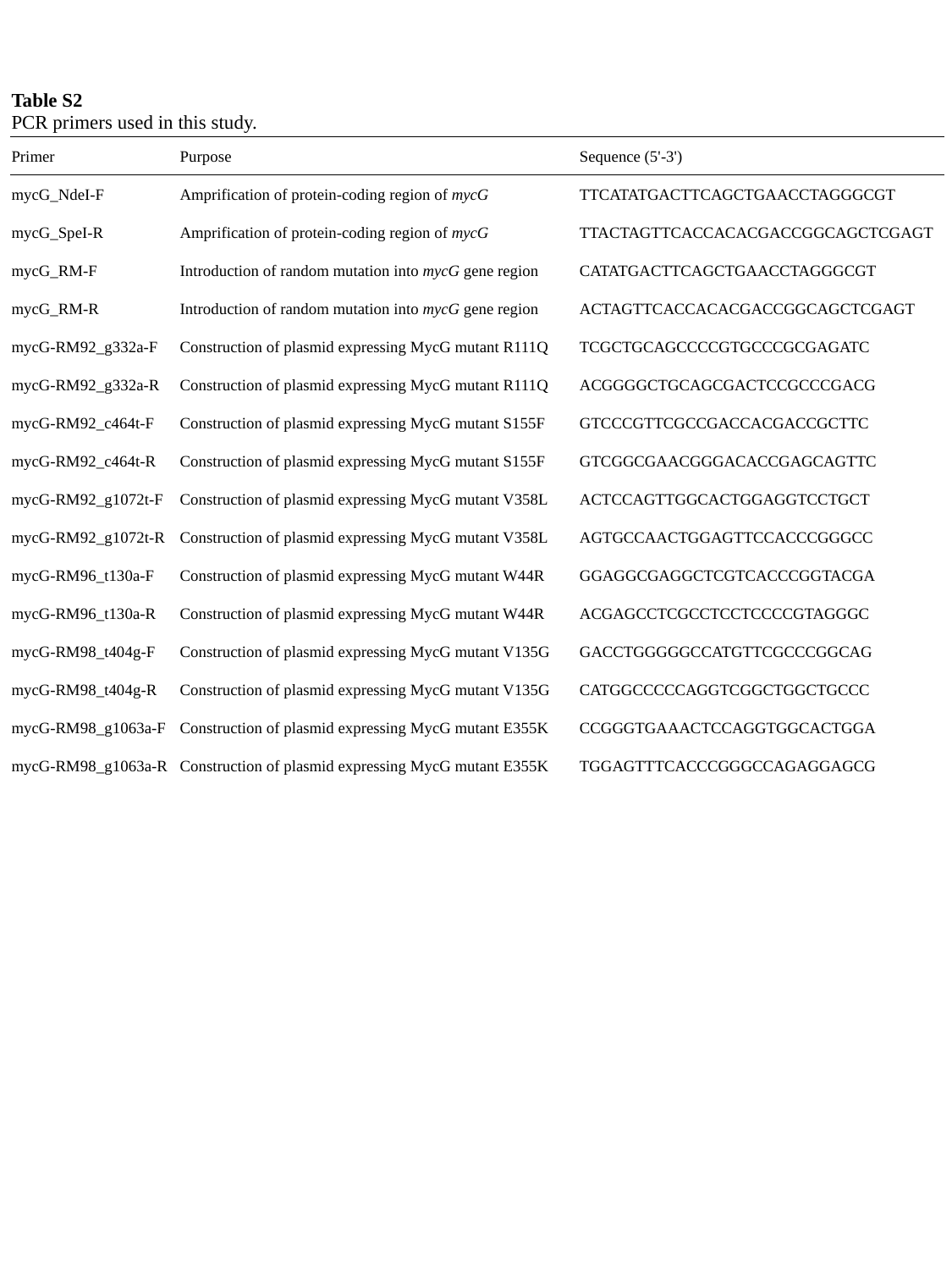

| Table S2 PCR primers used in this study. | | |
| --- | --- | --- |
| Primer | Purpose | Sequence (5'-3') |
| mycG\_NdeI-F | Amprification of protein-coding region of mycG | TTCATATGACTTCAGCTGAACCTAGGGCGT |
| mycG\_SpeI-R | Amprification of protein-coding region of mycG | TTACTAGTTCACCACACGACCGGCAGCTCGAGT |
| mycG\_RM-F | Introduction of random mutation into mycG gene region | CATATGACTTCAGCTGAACCTAGGGCGT |
| mycG\_RM-R | Introduction of random mutation into mycG gene region | ACTAGTTCACCACACGACCGGCAGCTCGAGT |
| mycG-RM92\_g332a-F | Construction of plasmid expressing MycG mutant R111Q | TCGCTGCAGCCCCGTGCCCGCGAGATC |
| mycG-RM92\_g332a-R | Construction of plasmid expressing MycG mutant R111Q | ACGGGGCTGCAGCGACTCCGCCCGACG |
| mycG-RM92\_c464t-F | Construction of plasmid expressing MycG mutant S155F | GTCCCGTTCGCCGACCACGACCGCTTC |
| mycG-RM92\_c464t-R | Construction of plasmid expressing MycG mutant S155F | GTCGGCGAACGGGACACCGAGCAGTTC |
| mycG-RM92\_g1072t-F | Construction of plasmid expressing MycG mutant V358L | ACTCCAGTTGGCACTGGAGGTCCTGCT |
| mycG-RM92\_g1072t-R | Construction of plasmid expressing MycG mutant V358L | AGTGCCAACTGGAGTTCCACCCGGGCC |
| mycG-RM96\_t130a-F | Construction of plasmid expressing MycG mutant W44R | GGAGGCGAGGCTCGTCACCCGGTACGA |
| mycG-RM96\_t130a-R | Construction of plasmid expressing MycG mutant W44R | ACGAGCCTCGCCTCCTCCCCGTAGGGC |
| mycG-RM98\_t404g-F | Construction of plasmid expressing MycG mutant V135G | GACCTGGGGGCCATGTTCGCCCGGCAG |
| mycG-RM98\_t404g-R | Construction of plasmid expressing MycG mutant V135G | CATGGCCCCCAGGTCGGCTGGCTGCCC |
| mycG-RM98\_g1063a-F | Construction of plasmid expressing MycG mutant E355K | CCGGGTGAAACTCCAGGTGGCACTGGA |
| mycG-RM98\_g1063a-R | Construction of plasmid expressing MycG mutant E355K | TGGAGTTTCACCCGGGCCAGAGGAGCG |

## Slide 4
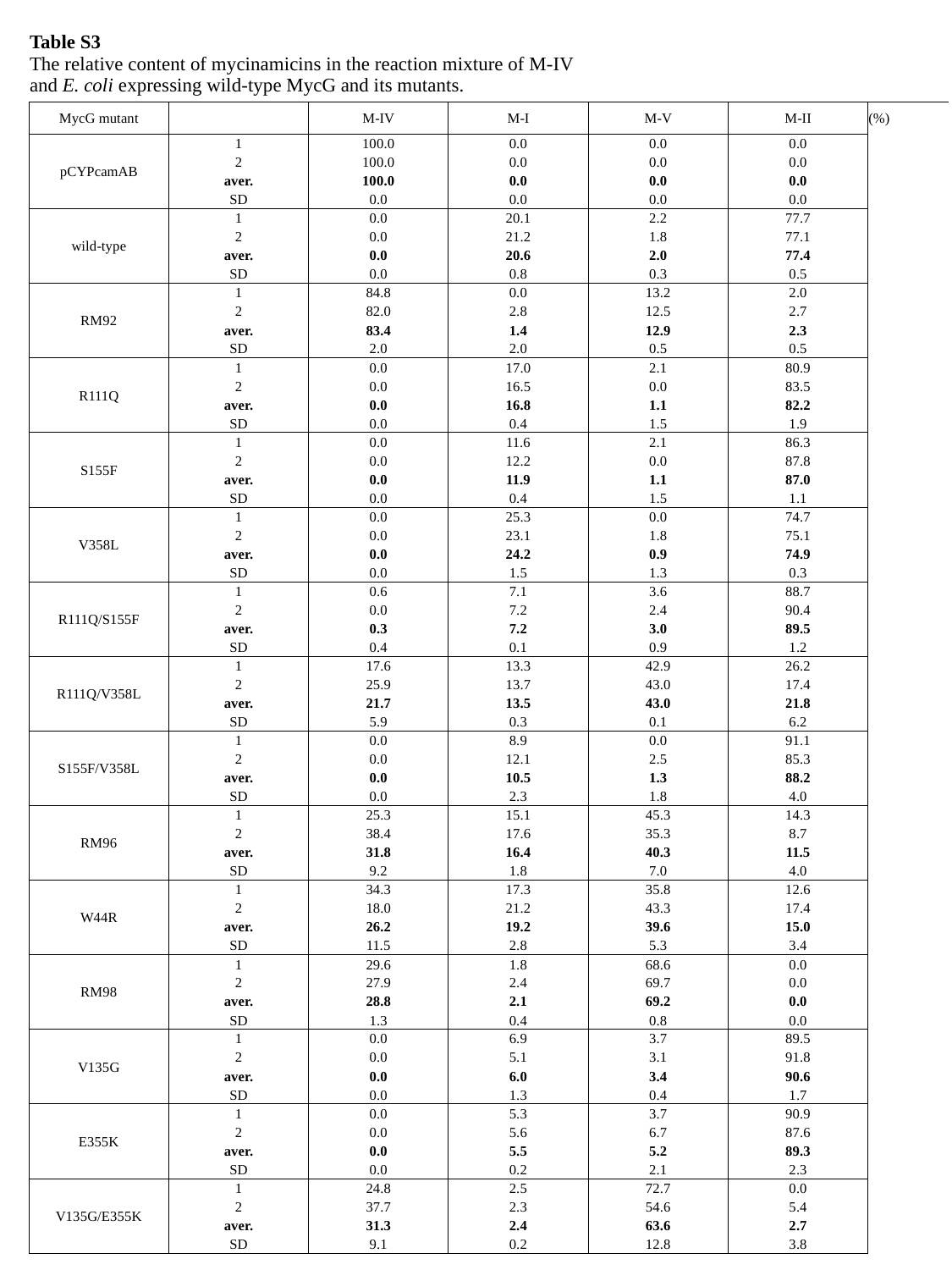

| Table S3 The relative content of mycinamicins in the reaction mixture of M-IV and E. coli expressing wild-type MycG and its mutants. | | | | | | |
| --- | --- | --- | --- | --- | --- | --- |
| MycG mutant | | M-IV | M-I | M-V | M-II | (%) |
| pCYPcamAB | 1 | 100.0 | 0.0 | 0.0 | 0.0 | |
| | 2 | 100.0 | 0.0 | 0.0 | 0.0 | |
| | aver. | 100.0 | 0.0 | 0.0 | 0.0 | |
| | SD | 0.0 | 0.0 | 0.0 | 0.0 | |
| wild-type | 1 | 0.0 | 20.1 | 2.2 | 77.7 | |
| | 2 | 0.0 | 21.2 | 1.8 | 77.1 | |
| | aver. | 0.0 | 20.6 | 2.0 | 77.4 | |
| | SD | 0.0 | 0.8 | 0.3 | 0.5 | |
| RM92 | 1 | 84.8 | 0.0 | 13.2 | 2.0 | |
| | 2 | 82.0 | 2.8 | 12.5 | 2.7 | |
| | aver. | 83.4 | 1.4 | 12.9 | 2.3 | |
| | SD | 2.0 | 2.0 | 0.5 | 0.5 | |
| R111Q | 1 | 0.0 | 17.0 | 2.1 | 80.9 | |
| | 2 | 0.0 | 16.5 | 0.0 | 83.5 | |
| | aver. | 0.0 | 16.8 | 1.1 | 82.2 | |
| | SD | 0.0 | 0.4 | 1.5 | 1.9 | |
| S155F | 1 | 0.0 | 11.6 | 2.1 | 86.3 | |
| | 2 | 0.0 | 12.2 | 0.0 | 87.8 | |
| | aver. | 0.0 | 11.9 | 1.1 | 87.0 | |
| | SD | 0.0 | 0.4 | 1.5 | 1.1 | |
| V358L | 1 | 0.0 | 25.3 | 0.0 | 74.7 | |
| | 2 | 0.0 | 23.1 | 1.8 | 75.1 | |
| | aver. | 0.0 | 24.2 | 0.9 | 74.9 | |
| | SD | 0.0 | 1.5 | 1.3 | 0.3 | |
| R111Q/S155F | 1 | 0.6 | 7.1 | 3.6 | 88.7 | |
| | 2 | 0.0 | 7.2 | 2.4 | 90.4 | |
| | aver. | 0.3 | 7.2 | 3.0 | 89.5 | |
| | SD | 0.4 | 0.1 | 0.9 | 1.2 | |
| R111Q/V358L | 1 | 17.6 | 13.3 | 42.9 | 26.2 | |
| | 2 | 25.9 | 13.7 | 43.0 | 17.4 | |
| | aver. | 21.7 | 13.5 | 43.0 | 21.8 | |
| | SD | 5.9 | 0.3 | 0.1 | 6.2 | |
| S155F/V358L | 1 | 0.0 | 8.9 | 0.0 | 91.1 | |
| | 2 | 0.0 | 12.1 | 2.5 | 85.3 | |
| | aver. | 0.0 | 10.5 | 1.3 | 88.2 | |
| | SD | 0.0 | 2.3 | 1.8 | 4.0 | |
| RM96 | 1 | 25.3 | 15.1 | 45.3 | 14.3 | |
| | 2 | 38.4 | 17.6 | 35.3 | 8.7 | |
| | aver. | 31.8 | 16.4 | 40.3 | 11.5 | |
| | SD | 9.2 | 1.8 | 7.0 | 4.0 | |
| W44R | 1 | 34.3 | 17.3 | 35.8 | 12.6 | |
| | 2 | 18.0 | 21.2 | 43.3 | 17.4 | |
| | aver. | 26.2 | 19.2 | 39.6 | 15.0 | |
| | SD | 11.5 | 2.8 | 5.3 | 3.4 | |
| RM98 | 1 | 29.6 | 1.8 | 68.6 | 0.0 | |
| | 2 | 27.9 | 2.4 | 69.7 | 0.0 | |
| | aver. | 28.8 | 2.1 | 69.2 | 0.0 | |
| | SD | 1.3 | 0.4 | 0.8 | 0.0 | |
| V135G | 1 | 0.0 | 6.9 | 3.7 | 89.5 | |
| | 2 | 0.0 | 5.1 | 3.1 | 91.8 | |
| | aver. | 0.0 | 6.0 | 3.4 | 90.6 | |
| | SD | 0.0 | 1.3 | 0.4 | 1.7 | |
| E355K | 1 | 0.0 | 5.3 | 3.7 | 90.9 | |
| | 2 | 0.0 | 5.6 | 6.7 | 87.6 | |
| | aver. | 0.0 | 5.5 | 5.2 | 89.3 | |
| | SD | 0.0 | 0.2 | 2.1 | 2.3 | |
| V135G/E355K | 1 | 24.8 | 2.5 | 72.7 | 0.0 | |
| | 2 | 37.7 | 2.3 | 54.6 | 5.4 | |
| | aver. | 31.3 | 2.4 | 63.6 | 2.7 | |
| | SD | 9.1 | 0.2 | 12.8 | 3.8 | |

## Slide 5
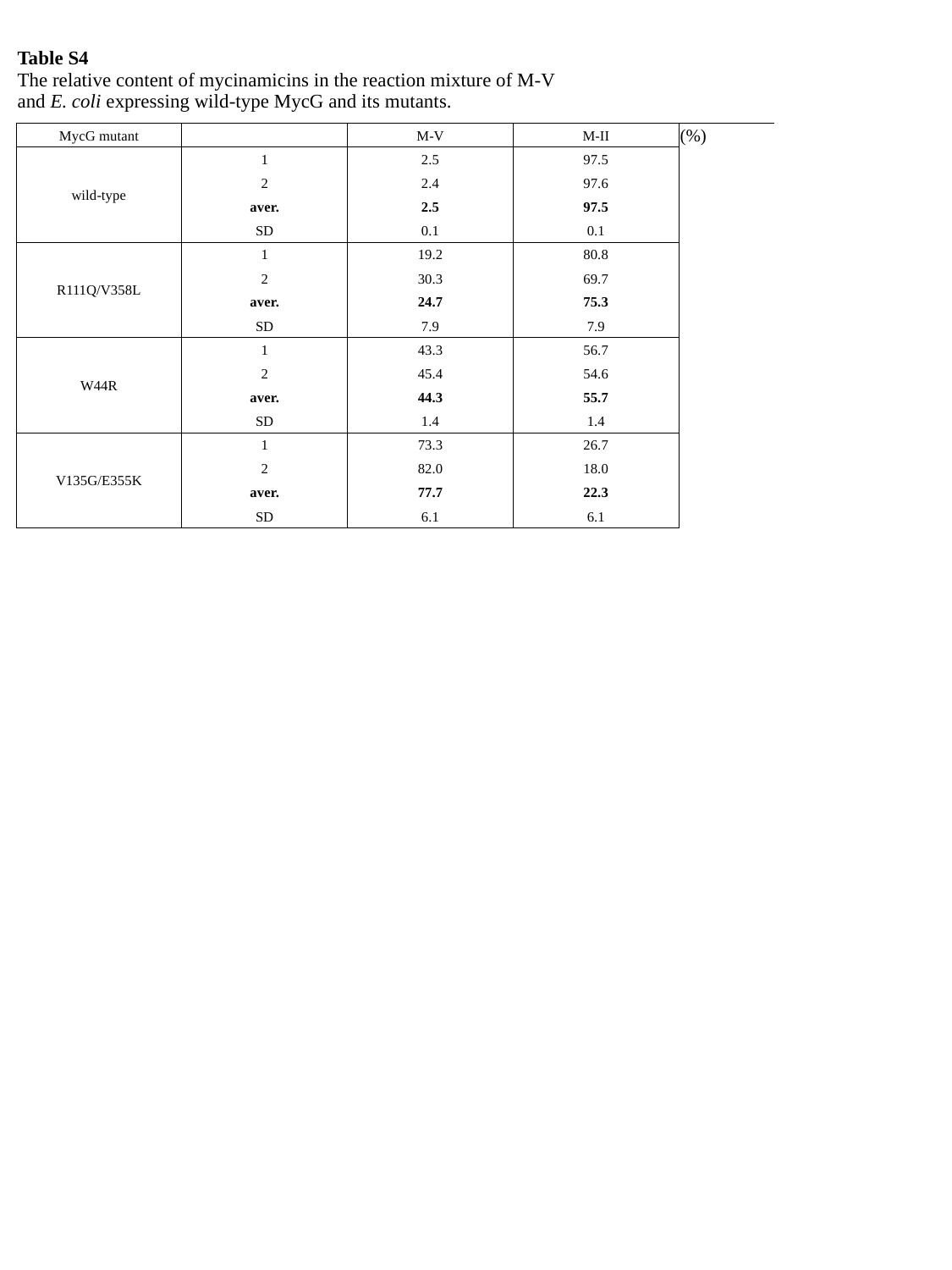

| Table S4 The relative content of mycinamicins in the reaction mixture of M-V and E. coli expressing wild-type MycG and its mutants. | | | | |
| --- | --- | --- | --- | --- |
| MycG mutant | | M-V | M-II | (%) |
| wild-type | 1 | 2.5 | 97.5 | |
| | 2 | 2.4 | 97.6 | |
| | aver. | 2.5 | 97.5 | |
| | SD | 0.1 | 0.1 | |
| R111Q/V358L | 1 | 19.2 | 80.8 | |
| | 2 | 30.3 | 69.7 | |
| | aver. | 24.7 | 75.3 | |
| | SD | 7.9 | 7.9 | |
| W44R | 1 | 43.3 | 56.7 | |
| | 2 | 45.4 | 54.6 | |
| | aver. | 44.3 | 55.7 | |
| | SD | 1.4 | 1.4 | |
| V135G/E355K | 1 | 73.3 | 26.7 | |
| | 2 | 82.0 | 18.0 | |
| | aver. | 77.7 | 22.3 | |
| | SD | 6.1 | 6.1 | |

## Slide 6
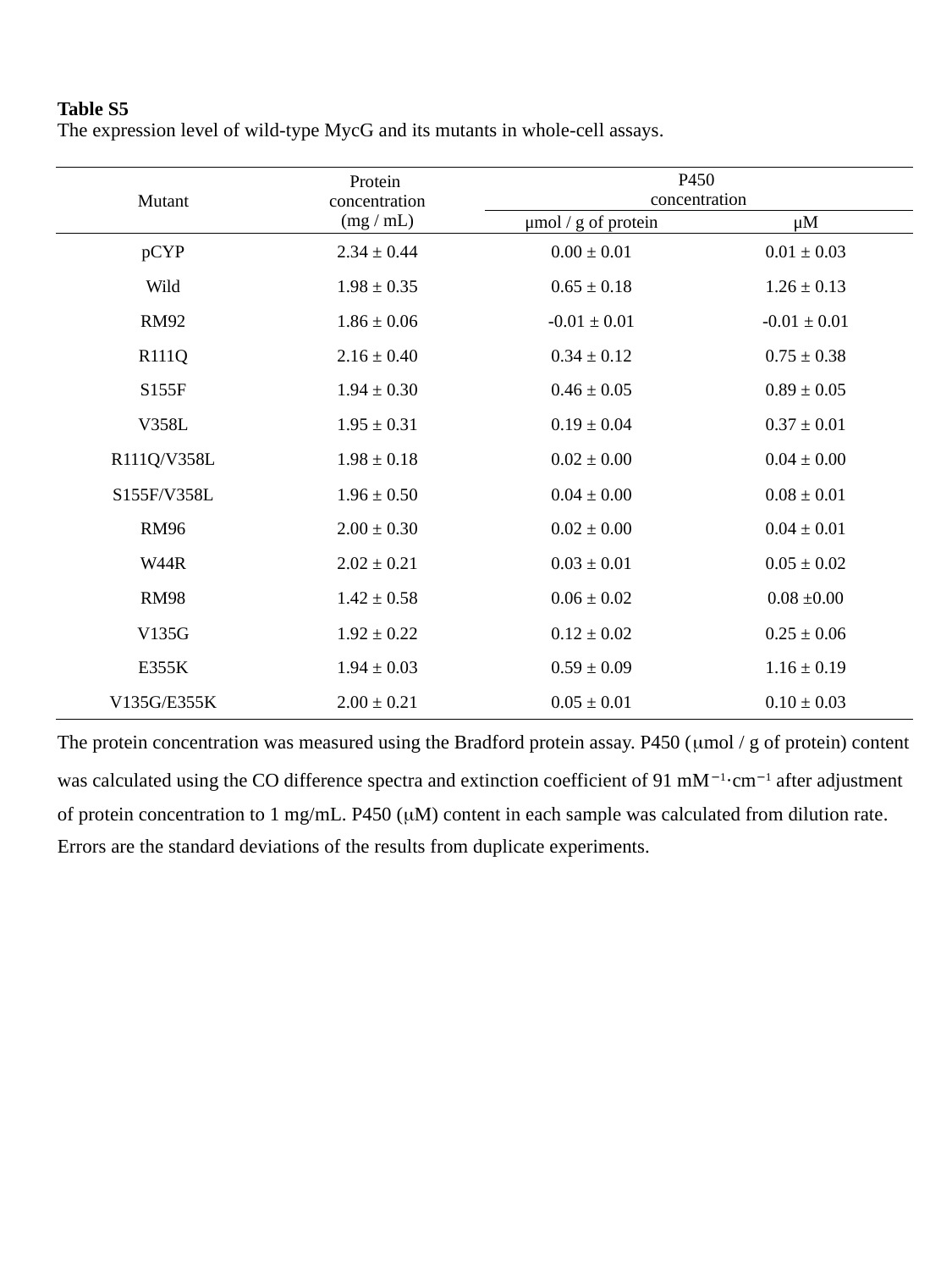

| Table S5 The expression level of wild-type MycG and its mutants in whole-cell assays. | | | |
| --- | --- | --- | --- |
| Mutant | Protein concentration (mg / mL) | P450 concentration | |
| | | μmol / g of protein | μM |
| pCYP | 2.34 ± 0.44 | 0.00 ± 0.01 | 0.01 ± 0.03 |
| Wild | 1.98 ± 0.35 | 0.65 ± 0.18 | 1.26 ± 0.13 |
| RM92 | 1.86 ± 0.06 | -0.01 ± 0.01 | -0.01 ± 0.01 |
| R111Q | 2.16 ± 0.40 | 0.34 ± 0.12 | 0.75 ± 0.38 |
| S155F | 1.94 ± 0.30 | 0.46 ± 0.05 | 0.89 ± 0.05 |
| V358L | 1.95 ± 0.31 | 0.19 ± 0.04 | 0.37 ± 0.01 |
| R111Q/V358L | 1.98 ± 0.18 | 0.02 ± 0.00 | 0.04 ± 0.00 |
| S155F/V358L | 1.96 ± 0.50 | 0.04 ± 0.00 | 0.08 ± 0.01 |
| RM96 | 2.00 ± 0.30 | 0.02 ± 0.00 | 0.04 ± 0.01 |
| W44R | 2.02 ± 0.21 | 0.03 ± 0.01 | 0.05 ± 0.02 |
| RM98 | 1.42 ± 0.58 | 0.06 ± 0.02 | 0.08 ±0.00 |
| V135G | 1.92 ± 0.22 | 0.12 ± 0.02 | 0.25 ± 0.06 |
| E355K | 1.94 ± 0.03 | 0.59 ± 0.09 | 1.16 ± 0.19 |
| V135G/E355K | 2.00 ± 0.21 | 0.05 ± 0.01 | 0.10 ± 0.03 |
| The protein concentration was measured using the Bradford protein assay. P450 (mmol / g of protein) content was calculated using the CO difference spectra and extinction coefficient of 91 mM−1·cm−1 after adjustment of protein concentration to 1 mg/mL. P450 (mM) content in each sample was calculated from dilution rate. Errors are the standard deviations of the results from duplicate experiments. | | | |

## Slide 7
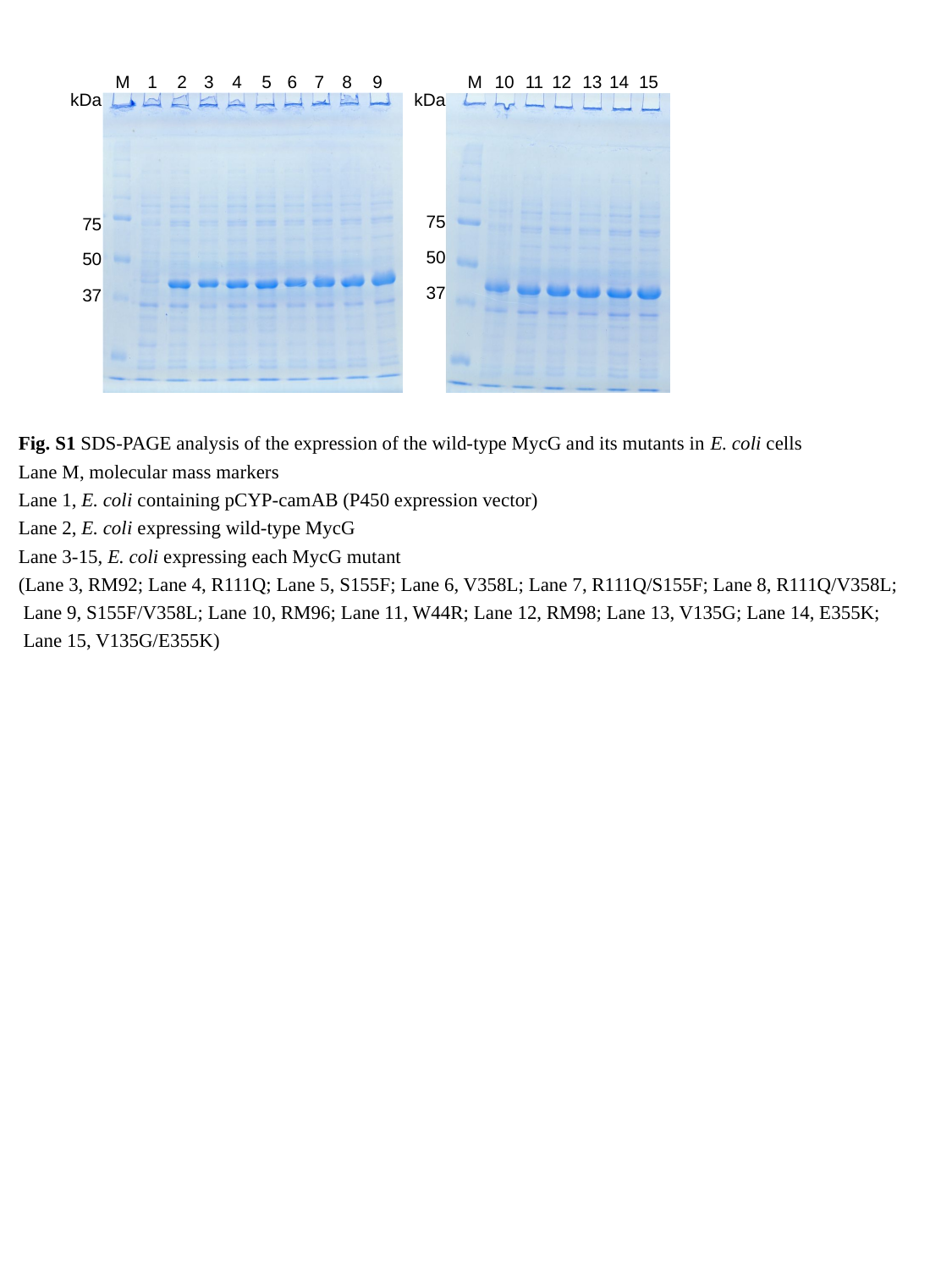

M
1
2
3
4
5
6
7
8
9
M
10
11
12
13
14
15
kDa
kDa
75
75
50
50
37
37
Fig. S1 SDS-PAGE analysis of the expression of the wild-type MycG and its mutants in E. coli cells
Lane M, molecular mass markers
Lane 1, E. coli containing pCYP-camAB (P450 expression vector)
Lane 2, E. coli expressing wild-type MycG
Lane 3-15, E. coli expressing each MycG mutant
(Lane 3, RM92; Lane 4, R111Q; Lane 5, S155F; Lane 6, V358L; Lane 7, R111Q/S155F; Lane 8, R111Q/V358L;
 Lane 9, S155F/V358L; Lane 10, RM96; Lane 11, W44R; Lane 12, RM98; Lane 13, V135G; Lane 14, E355K;
 Lane 15, V135G/E355K)

## Slide 8
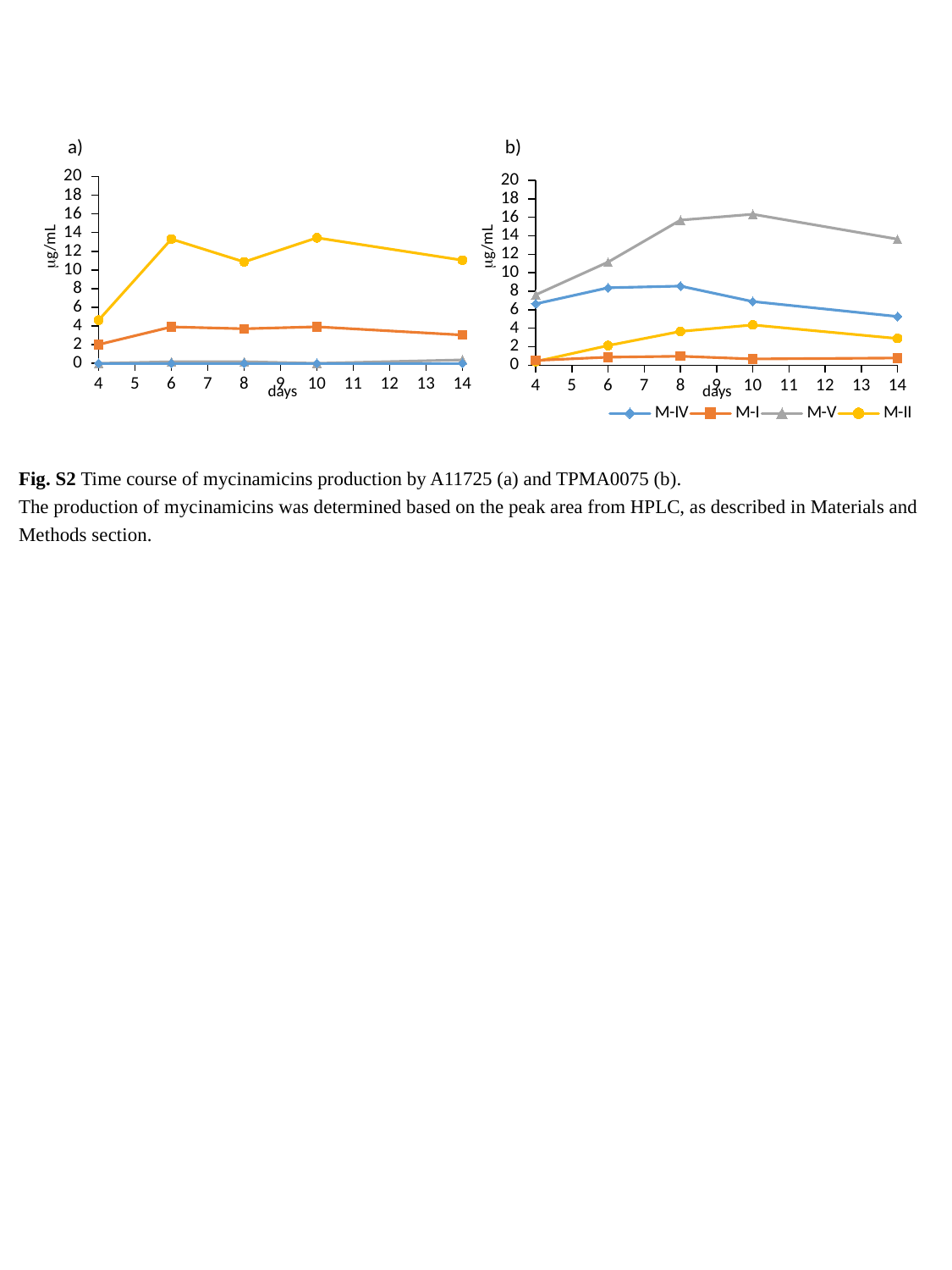

b)
a)
### Chart
| Category | M-IV | M-I | M-V | M-II |
|---|---|---|---|---|
### Chart
| Category | M-IV | M-I | M-V | M-II |
|---|---|---|---|---|mg/mL
mg/mL
days
days
Fig. S2 Time course of mycinamicins production by A11725 (a) and TPMA0075 (b).
The production of mycinamicins was determined based on the peak area from HPLC, as described in Materials and Methods section.
